# Partial characterization of two novel monoclonal antibodies for *Listeria *spp. Immunodetection

**DOI:** 10.1186/1753-6561-8-S4-P64

**Published:** 2014-10-01

**Authors:** Gustavo Moreira, Marcelo Mendonça, Fabricio Conceição

**Affiliations:** 1 Centro de Desenvolvimento Tecnológico, Núcleo de Biotecnologia, Universidade Federal de Pelotas, Pelotas, Rio Grande do Sul, 96010-900, Brazil

## Background

The *Listeria *genus comprises ten different species, however, only two of them - *L. monocytogenes *and *L. ivanovii *- are pathogenic. Although being a well-known pathogen, its detection methodologies are still limited. The gold-standard technique involves enrichment, isolation, and biochemical characterization, which can take about 7 days [1]. In addition, since both pathogenic and non-pathogenic species are present in contaminated food, it is important to detect them both [2]. This way, new methods, such as the immunodetection using monoclonal antibodies (MAbs), have been developed to reduce the detection time of *Listeria *spp.. Recently, our group described a hybridoma clone secreting a MAb of the IgM isotype, called MAb-3F8, that reacts exclusively with an antigen of 30 kDa (P30) present in all *Listeria *spp. evaluated [3]. In the present work, we produced and initially characterized, by ELISA and Western blot, other two novel antibodies against P30 to be used in *Listeria *spp. immunodetection assays.

## Materials and methods

A female BALB/c mouse was initially inoculated intraperitoneally with 100 µg of recombinant P30 (rP30) with complete Freund's Adjuvant (FA). Fifteen days after the first injection, a series of 13 doses of rP30 with incomplete FA were performed weekly. Then, the mouse splenocytes were obtained and further fused with Sp2/O cells. The resulting hybridomas were cultured and screened by ELISA using both rP30 and formalin-killed *L. monocytogenes *strain ATCC 19117 (Lm19117) as antigens. Thereby, two hybridomas were selected and used to produce ascites in mice [4]. MAbs isotyping kit (Sigma Aldrich) was used to determine the isotype of each MAb. For the ELISA, rP30 and formalin-killed Lm19117 and *L. innocua *(Linn) were used as antigens. For the Western blot (WB), Lm19117 cell-wall fraction [5] and rP30 were transferred to a PVDF membrane. In both assays, the ascites and anti-mouse/peroxidase (Sigma Aldrich) were primary and secondary antibodies, respectively. In addition, 3F8 (positive control) and a non-immunized mouse serum (negative control) were used. Absorbance results at least 3 times higher than the negatives were considered positives.

## Results and conclusions

The isotyping assay determined that one MAb was IgG1 (MAb-G2A) and the other, IgM (MAb-M5A). In the ELISA, MAb-G2A reacted with rP30 (OD_450_=0.346), but not with Lm19117 (OD_450_=0.020) or Linn (OD_450_=0.018). However, M5A reacted with rP30 (OD_450_=0.160), Lm19117 (OD_450_=0.229), and Linn (OD_450_=0.199). Negatives for rP30, Lm, and Linn were 0.026, 0.048, and 0.062, respectively; while the positives were 0.418, 0.237, and 0.211, respectively. In the WB, the same pattern seem to occur with MAb-G2A, since it reacted only with rP30; while MAb-M5A presented no reaction. The fact that M5A had no reaction in WB, but showed promising results in ELISA indicate this MAb binds to a conformational epitope of P30 that is displayed on *Listeria *surface. Considering this, further studies will be conducted with a larger panel of bacteria to better characterize these MAbs and determine their sensibility and specificity in detecting *Listeria *spp. in food samples.
